# Novel Recombinant Sapovirus, Japan

**DOI:** 10.3201/eid1205.051608

**Published:** 2006-05

**Authors:** Tung Gia Phan, Shoko Okitsu, Werner E.G. Müller, Hideki Kohno, Hiroshi Ushijima

**Affiliations:** * University of Tokyo, Tokyo, Japan;; † Institut für Physiologische Chemie, Mainz, Germany;; ‡ Nihon University, Chiba, Japan

**Keywords:** Sapovirus, recombination, Japan, letter

**To the Editor:** *Sapovirus* is the distinct genus within the family *Caliciviridae*; these viruses cause sporadic cases and outbreaks of gastroenteritis in humans worldwide (*1*). The sapovirus genome contains 2 open reading frames (ORFs). ORF1 encodes nonstructural and capsid proteins while ORF2 encodes a small protein (*2*). Sapovirus has a typical "Star of David" configuration by electron microscopic examination. The prototype sapovirus is the Sapporo virus (Hu/SaV/Sapporo virus/1977/JP), which was originally discovered from an outbreak in a home for infants in Sapporo, Japan, in 1977 (*3*). Sapovirus is divided into 5 genogroups, among which only genogroups I, II, IV, and V are known to infect humans (*4*).

A fecal specimen was collected from a 1-year-old boy with acute gastroenteritis in Osaka, Japan, in March 2005. The viral genome was extracted by using a QIAamp kit (Quigen, Hilden, Germany). By using multiplex reverse transcription–polymerase chain reaction (RT-PCR), 2 groups of diarrheal viruses were identified. The first group included astrovirus, norovirus, and sapovirus; the second group included rotavirus and adenovirus (*5*). Sapovirus polymerase region was also amplified to identify recombinant sapovirus by using primers P290 and P289 (*6*). To eliminate the possibility of co-infection of 2 different sapovirus genotypes, to localize the potential recombination site, and to understand a possible recombination mechanism of recombinant sapovirus, flanking polymerase and capsid regions, with their junction of HU/5862/Osaka/JP, were amplified with primers P290 and SLV5749 to produce a 1,162-bp product (*5**,**6*). Products were directly sequenced, and capsid- and polymerase-based phylogenetic trees showed recombinant sapovirus.

The fecal specimen was positive for sapovirus. HU/5862/Osaka/JP clustered into the genogroup I genotype 8 (GI/8 the 8/DCC/Tokyo/JP/44 cluster) (Figure) by using the recent sapovirus capsid region classification (*7*). HU/5862/Osaka/JP with GI/8 capsid was classified into GI/1 (the Sapporo/82 cluster) when polymerase-based grouping was performed. When the sequence of HU/5862/Osaka/JP was compared with that of Sapporo/82 by using SimPlot Version 1.3 (available from http://sray.med.som.jhmi.edu/SCRoftware/simplot), the recombination site was identified at the polymerase-capsid junction. Before this junction, sequences of HU/5862/Osaka/JP and Sapporo/82 were highly homologous. However, homology between them was notably different after the junction, with a sudden drop in the identity for HU/5862/Osaka/JP. By using ClustalX, HU/5862/Osaka/JP shared a 96% identity in polymerase sequence and an 85% identity in capsid sequence with Sapporo/82. In contrast, homology was 99% in the capsid region between HU/5862/Osaka/JP and 8/DCC/Tokyo/JP/44. Since a polymerase sequence of 8/DCC/Tokyo/JP/44 was not available in GenBank because of the unsuccessful amplification, homology in the polymerase region between HU/5862/Osaka/JP and 8/DCC/Tokyo/JP/44 was unknown.

**Figure:**
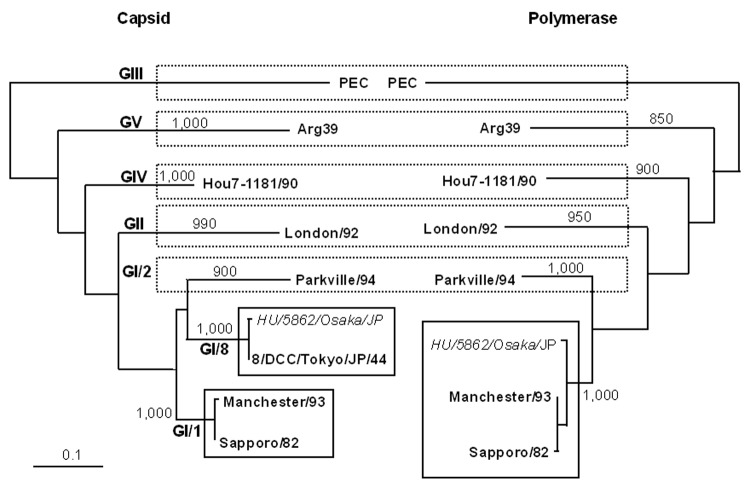
Changing genotypes of sapovirus on the basis of phylogenetic trees. Trees were constructed from partial amino acid sequences of capsid and polymerase of HU/5862/Osaka/JP highlighted in *italics*. Phylogenetic tree with 1,000 bootstrap resamples of the nucleotide alignment datasets was generated by using the neighbor-joining method with ClustalX. The genetic distance was calculated by using Kimura 2-parameter method (PHYLIP). The scale indicates amino acid substitutions per position. The numbers in branches indicate bootstrap values. Porcine enteric calicivirus was used as an outgroup strain for phylogenetic analysis. The nucleotide sequence data of sapovirus strain HU/5862/Osaka/JP has been submitted to GenBank and has been assigned accession no. DQ318530. Reference sapovirus strains and accession nos. used in this study were as follows: PEC (AF182760), London/92 (U95645), Arg39 (AY289803), Parkville/94 (U73124), Manchester/93 (X86560), Sapporo/82 (U65427), Hou7-1181/90 (AF435814), and 8/DCC/Tokyo/Japan/44 (AB236377).

Altogether, the findings underscored that HU/5862/Osaka/JP represented a novel, naturally occurring, recombinant sapovirus with GI/8 capsid and GI/1 polymerase. To determine whether the child was infected with this novel recombinant sapovirus or whether the novel recombinant sapovirus resulted from co-infection with 2 different viruses, Svppo (Sapporo/82-specific primer), Svdcc (8/DCC/Tokyo/JP/44-specific primer), and SLV5749 were used to amplify the capsid region (*5*). However, no amplicon was found. These negative results indicate no co-infection in this child.

Even though many molecular epidemiologic studies on sapovirus infection have been performed worldwide, reports documenting recombination in sapovirus are still limited. The first recombinant sapovirus identified was the Thai isolate Mc10 or the Japanese isolate C12 (*8*); the Japanese isolate Ehime1107 and the SW278 isolate from Sweden were identified later (*9*). Recombination occurred only in sapovirus genogroup II, which is more capable of recombination than other genogroups (*8**,**9*). In this study, we identified HU/5862/Osaka/JP with a novel recombination between 2 distinct genotypes within genogroup I. This is the first report of acute gastroenteritis caused by recombinant sapovirus genogroup I. The findings underscore that natural recombination occurs not only in sapovirus genogroup II but also in genogroup I.

In recent studies of sapovirus recombination, evidence for the location of the recombination event is lacking because of the distant geographic relationship of parent and progeny strains. HU/5862/Osaka/JP shared the closest sequences of polymerase and capsid with Sapporo/82 and 8/DCC/Tokyo/JP/44, respectively. Sapporo/82 was first isolated in 1982, and 8/DCC/Tokyo/JP/44 was isolated in 2000, both in Japan. Possibly, Sapporo/82 and 8/DCC/Tokyo/JP/44 were parental strains of HU/5862/Osaka/JP, and the event leading to the novel recombination might have occurred in Japan.

The capsid region was used for genotype classification of sapovirus (*7*). When capsid-based grouping was performed, HU/5862/Osaka/JP distinctly belonged to genotype 8. When polymerase-based grouping was performed, HU/5862/Osaka/JP distinctly belonged to genotype 1. Therefore, sapovirus classification based on capsid sequence is questionable. We suggest that sapovirus classification should rely not only on capsid sequence but also on polymerase sequence.
